# The relationship between relative solvent accessible surface area (rASA) and irregular structures in protean segments (ProSs)

**DOI:** 10.6026/97320630012381

**Published:** 2016-11-28

**Authors:** Divya Shaji

**Affiliations:** 1 Graduate School of Information Science, Nagoya University, Furo-cho, Chikusa-ku, Nagoya 464-8601, Japan

**Keywords:** Intrinsically disordered proteins, secondary structure elements (SSEs), protein interface, relative solvent accessible surface area (rASA), protein-protein interactions, protean segments (ProSs)

## Abstract

Intrinsically Disordered Proteins (IDPs) lack a stable, three-dimensional structure under physiological conditions, yet they exhibit numerous biological activities. Protean segments (ProSs) are the functional regions of intrinsically disordered proteins that undergo disorder-to-order transitions upon binding to their partners. Example ProSs collected from the intrinsically disordered proteins with extensive annotations and literature (IDEAL) database. The interface of protean segments (ProSs) is classified into core, rim, and support, and analyzed their secondary structure elements (SSEs) based on the relative accessible surface area (rASA). The amino acid compositions and the relative solvent accessible surface areas (rASAs) of ProS secondary structural elements (SSEs) at the interface, core and rim were compared to those of heterodimers. The average number of contacts of alpha helices and irregular residues was calculated for each ProS and heterodimer. Furthermore, the ProSs were classified into high and low efficient based on their average number of contacts at the interface. The results indicate that the irregular structures of ProSs and heterodimers are significantly different. The rASA of irregular structures in the monomeric state (rASAm) is large, leads to the formation of larger ΔrASA and many contacts in ProSs.

## Background

An intrinsically disordered protein (IDP) is a protein that is disordered (as a whole or in part) in the unbound state and undergoes a disorder-to-order transition upon binding to their partners [1,2,3]. These IDPs have numerous biological activities such as signal transduction and transcriptional regulation and are highly abundant in nature [2,4]. These proteins are associated with various human diseases, including cancer, cardiovascular disease, neurodegenerative diseases and amyloidoses [5,6,7]. Due to their role in various biological processes and their involvement in various diseases, IDPs are the focus of many biomedical-related areas and represent attractive novel drug targets [7,8].

Protean segments (ProSs) are the functional regions of intrinsically disordered proteins that undergo disorder-to-order transitions upon binding to their partners (i.e., coupled folding and binding) [9,10, 11,12]. The ProS interface is composed of a small core and a large rim. The average number of contacts of ProS interface with its interaction partners is greater than that of heterodimers. This indicates the effective interactions of ProSs that take place in the rim region like core. The key to effective interactions of ProSs is the solvent exposure of rim residues in the monomeric state (rASAm) [13].

The goal of this work is to investigate the properties of secondary structure elements (SSEs) at the interface of ProSs relative to those of heterodimers. The interfaces of ProSs and heterodimers were classified into the core, rim, and support based on their relative solvent accessible surface area (rASA) [14]. The average number of contacts of alpha helices and irregular residues was calculated for each ProS and heterodimer. Furthermore, the ProSs were classified into high and low efficient ProSs based on their average number of contacts at the interface. Compared to heterodimers, irregular residues of ProSs have larger number of contacts than their alpha helices. Moreover, irregular residues of ProSs have larger ΔrASA than their alpha helices. The rASA of irregular structures in the monomeric state is large, that leads to the formation of larger ΔrASA and many contacts in ProSs. In addition, high efficient ProSs have larger average rASA in the monomeric state (rASAm) and larger average ΔrASA, than low efficient ProSs.

## Materials and Methods

### ProSs and heterodimers

All ProSs (210) in 70 protein sequences were collected from the IDEAL database (as of August 2013) [11,12]. If more than one ProS were found in a protein and their positions overlapped, the longest ProS was selected. The sequence redundancy was removed with 80% sequence similarity (based on the CLUSTALW alignment) [15]. Hierarchical clustering was done with R [16] using completelinkage clustering and the longest ProS in a cluster was selected as the representatives. A non-redundant set contained 99 ProSs [13]. DNA-binding ProSs and one-to-many binding ProSs (a single ProS binds to two or more different partners), were discarded [17]. Both the X-ray and NMR structures were used in this study.

A non-redundant dataset of 276 heterodimers was selected from the Protein Data Bank (PDB) [18], using the PDB’s advanced search interface (as of July 2014). The search criteria satisfied the following conditions: (1) less than 30% sequence identity; (2) the macromolecule type contained only proteins; (3) the oligomeric state was heterodimer; (4) each chain was greater than 100 residues; and (5) structures determined by X-ray crystallography had higher than 3 Å resolutions. Only smaller protomers were analyzed as the reference of ProSs.

### Secondary structure analysis

The program DSSP [19] was used to assign secondary structures. The eight types calculated by DSSP were reduced to three, such as alpha helices (H, G and I), beta strands (E) and irregulars (B, S, T and C). The amino acid propensity, average number of contacts and relative solvent accessible surface areas (rASAs) of alpha helices and irregulars were analyzed in detail.

### Calculation of amino acid propensities

The propensities of amino acids are represented as the Chou– Fasman parameters [20], CF (a,P) = Na(P)/N(P)/(Naall/Nall), where Na (P) is the number of amino acid residue a in place P, N (P) is the total number of residues in P, Naall is the total number of amino acid residue a in the protein sequence, and Nall is the total number of residues in the protein sequence. In P, the alpha helix and irregular residues of ProSs and heterodimers were considered. To calculate the reference states (the denominator), the same secondary structure types of PDBSelect25 [21] proteins were used. PDBSelect25 contains a representative set of PDB entries with less than 25 % sequence identity.

### Analysis of relative ASA (rASA) and residue contacts

The interfaces of each ProSs and heterodimer were classified into the core, rim and support based on the definitions of Levy [14]. The relative solvent accessible surface area (rASA) of a residue indicates a degree of residue solvent exposure. It can be calculated by normalizing the total accessible surface area (ASA) of the residues in a protein structure by the ASA of the residues in the most exposed state to a solvent molecule [22]. The program Naccess [23], which is an implementation of Lee and Richard’s algorithm [24] were used to calculate the rASA of each residue in the monomeric (rASAm) and complex states (rASAc) for ProSs and heterodimers. The change in relative solvent accessible surface area (ΔrASA) of each residue was calculated as the difference between the rASAs of monomeric (rASAm) and complex states (ASAc). The rASAs were averaged for the interface, core and rim residues, to derive the average rASAs of proteins.

Two residues, i and j, were considered to be in contact if any atom of residue i was within a distance of < 4.5 Å with any atom of residue j [25,26]. The average number of external contacts and relative solvent accessible surface areas (rASAs) at the interface, core and rim in alpha helices and irregular residues were calculated for each ProS and heterodimer. External contacts are defined as the contacts between the proteins and their interaction partners. The support and beta strand residues were discarded from this study because of their shortage in ProSs.

### High and low efficient ProSs

Based on the average number of contacts in the interface, the ProSs were classified into high and low efficient ProSs. High and low efficient ProSs were defined as the contacts of ProSs with greater than 4 and less than 2.5, respectively. Short ProSs (less than 11 residues) were discarded from this classification. Several properties were analyzed for each high and low efficient ProSs (See Results and Discussion). The datasets contain 11 and 14 ProSs for high and low efficient, respectively [13]. The radius of gyration was calculated using Bio3D package [27] in R [16].

### Statistical analysis

Wilcoxon rank-sum test was performed by RStudio [28] to calculate the P-values. P < 0.01 was considered statistically significant.

## Results and Discussion

### Secondary structure analysis of ProSs and heterodimers

**Figure 1:**
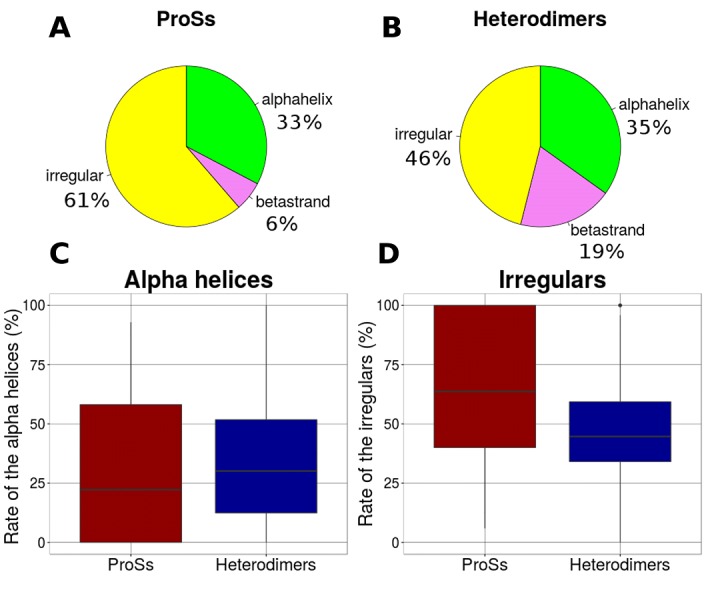
Distribution of secondary structure elements (SSEs) in ProS and heterodimer interface. The composition of secondary structure elements (SSEs) in ProS interface (A) and heterodimer interface (B). The program DSSP was used to assign secondary structures. The eight types calculated by DSSP [19] were reduced to three, such as alpha helices, beta strands, and irregulars. The distributions of alpha helices, beta strands and irregulars are colored in green, violet and yellow, respectively. Because of the shortage of beta strand residues in ProSs, alpha helices and irregulars were considered for further analysis. Box-plots of the rates of (C) alpha helix residues in ProSs (red) and heterodimers (blue) interface (D) irregular residues in ProSs and heterodimers interface. The distribution of the irregulars is significantly different as assessed by the Wilcoxon rank-sum test (alpha helices = 0.03, irregulars = 1.05e-07).

The secondary structure assignments for each of the ProS and heterodimer interface were determined by the DSSP program [19]. This analysis (See Figure 1A,B) showed that 33% of the residues in the ProSs dataset were alpha helices, 6% were beta strands, and 61% were residues of the irregular structure. The secondary structure distribution of ProSs interface is very different from those of heterodimers. The content of irregular structures and beta strands are the largest difference between ProSs and heterodimers. Alpha helices are almost equally abundant in both data sets. ProS interface contains 15% more irregular residues, 13% fewer beta strands and 2% fewer alpha helices than heterodimers. The differences between the distributions were evaluated, and the boxplots of the rates of alpha helices and irregulars are shown in Figure 1C and D. The alpha helix residues of ProSs and heterodimers are not significantly different (P-value = 0.03). It is important to note that, the irregular structures of ProSs and heterodimers are significantly different (P-value = 1.05e-07).

### Interactions of secondary structure elements (SSEs)

**Figure 2:**
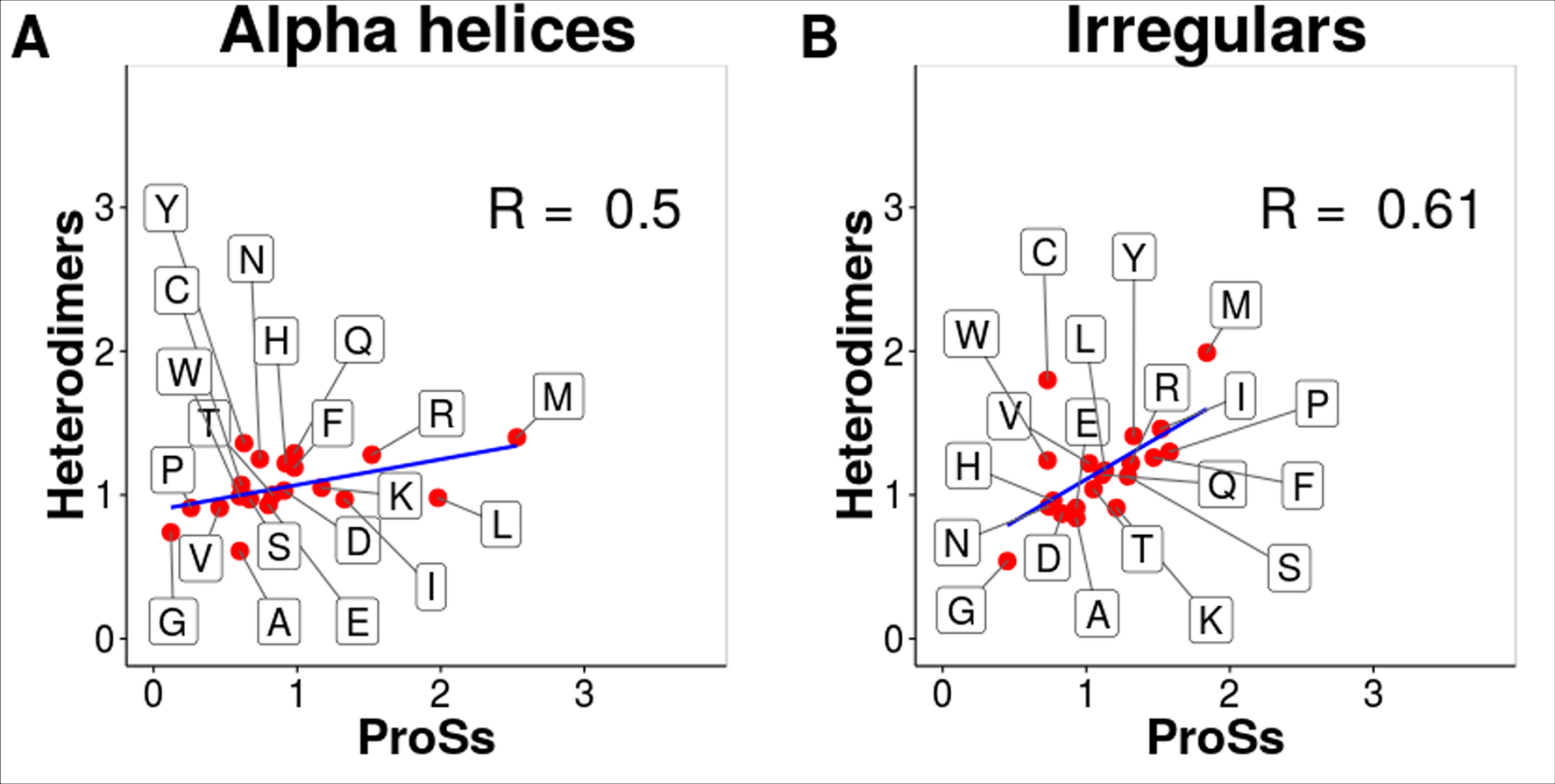
Scatter plots of the Chau–Fasman parameters [20] of alpha helices and irregulars at the interface (A) ProS alpha helices vs. heterodimer alpha helices. (B) ProS irregulars vs. heterodimer irregulars.

The amino acid propensities of the different secondary structure elements (SSEs) (alpha helices and irregular structures) for ProSs vs. heterodimers were examined. The Chou–Fasman parameters [20] for alpha helix and irregular residues at the interface were calculated. In Figure 2A and B, the correlations between ProS alpha helices vs. heterodimer alpha helices and ProS irregulars vs. heterodimer irregulars at the interface are indicated. In both cases, positive correlations were observed with 0.50 and 0.61 for alpha helix and irregular residues, respectively. This indicates that the amino acid composition of the ProSs secondary structural elements (SSEs) is moderately similar to that of heterodimers.

The core residues at the interface are the hydrophobic residues, generally in the central region of the interface, and play an important role in the interaction. The rim residues are the polar residues, located on the outer edges of the interface. The support residues represent the intersection between the interior and the interface [14].

**Figure 3:**
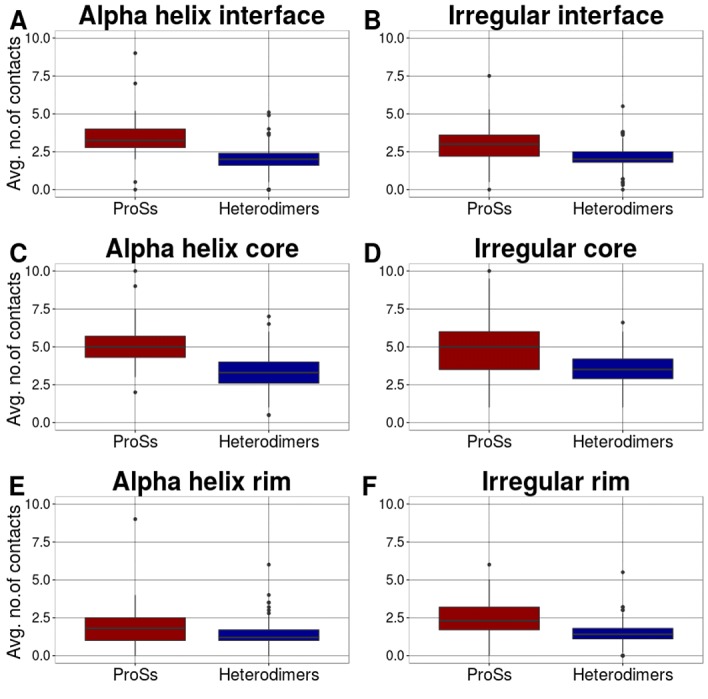
Interactions of the secondary structure elements (SSEs) in ProSs and heterodimers. Box-plots of the average number of contacts of the alpha helices and irregulars in ProSs and heterodimers at the interface (A and B), core (C and D) and rim (E and F). The distributions of ProSs and heterodimers are colored in red and blue, respectively (these colors are used throughout this paper). The differences between the distributions were evaluated, and the P-values are shown in Table 1 and 2.

Previous studies have been indicated that the ProS interface can be in contact with a larger number of residues of the interaction partners compared with the heterodimer interface [13,29]. To examine the efficiency of interactions in different secondary structural elements (SSEs), the average number of external contacts of the interface, core, and rim residues were calculated for each ProS and heterodimer (see Figure 3 A-F). Compared to heterodimers, irregular residues of ProSs have a larger number of contacts than their alpha helices. In Table 1 and Table 2, the P-values of alpha helices and irregulars are shown respectively, for the interface, core, and rim.

### Relative ASA (rASA) of secondary structure elements (SSEs)

**Figure 4:**
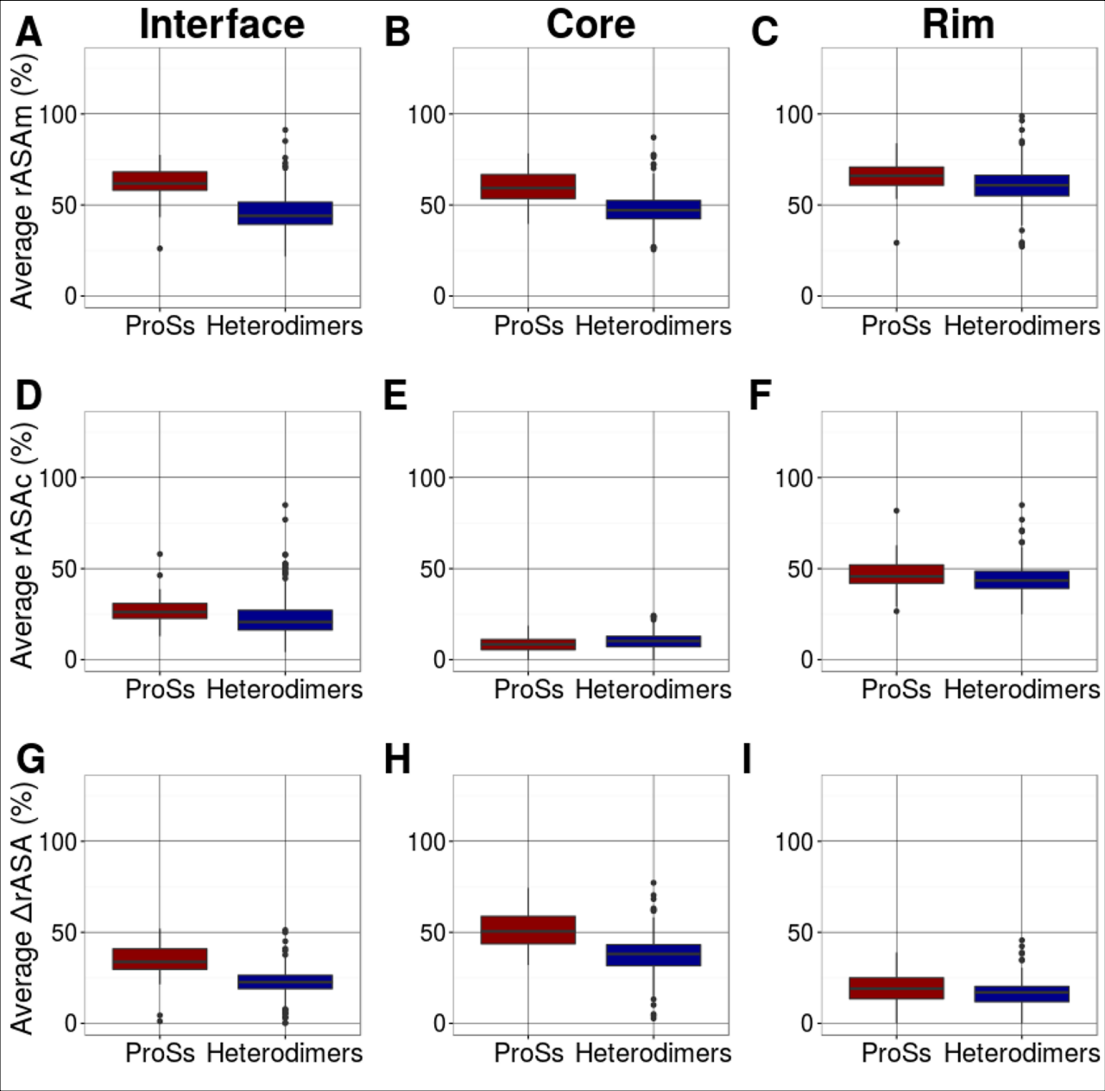
Average rASAs of alpha helices in ProSs and heterodimers. Average rASAm at the interface (A), core (B) and rim (C). Average rASAc at the interface (D), core (E) and rim (F). Average ΔrASA at the interface (G), core (H) and rim (I). The differences between the distributions were evaluated, and the Pvalues are shown in Table 1.

**Figure 5:**
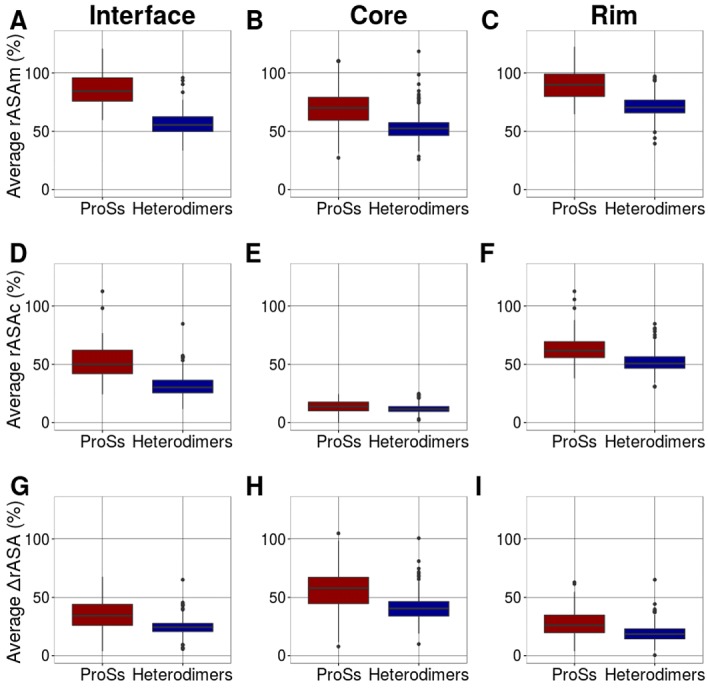
Average rASAs of irregulars in ProSs and heterodimers. Average rASAm at the interface (A), core (B) and rim (C). Average rASAc at the interface (D), core (E) and rim (F). Average ΔrASA at the interface (G), core (H) and rim (I). The differences between the distributions were evaluated, and the P-values are shown in Table 2.

Our previous study showed that the average ΔrASA correlates well with the average number of contacts in ProSs [13]. ΔrASA of each residue is defined by the difference between rASA of the unbound state (rASAm) and that of the bound state (rASAc), and both rASAs are used to define the core, rim and support residues (ΔrASA = rASAm -rASAc) [14]. Here, the relative solvent accessible surface areas (rASAs) of the alpha helices and irregular structures in each ProS and heterodimer at the interface, core and rim were analyzed in detail. In Figure 4 A-C, D-F, and G-I, the distribution of the average rASAm, rASAc and ΔrASA of ProS alpha helices is shown respectively, for the interface, core, and rim, and compared with those of heterodimers. Similarly, in Figure 5 A-C, D-F and G-I, the distribution of the average rASAm, rASAc and ΔrASA of ProS irregulars is shown respectively, for the interface, core, and rim, and compared with those of heterodimers. In both the core and rim, irregular residues of ProSs have a larger rASA in the monomeric state than heterodimers. The differences are confirmed by a statistical test (See Table 1 and Table 2). The rASA of ProS irregular residues in the monomeric state (rASAm) is large, resulting in a larger ΔrASA, leads to the formation of many contacts. Contour plots of average rASAm and rASAc of alpha helices and irregular structures are shown in Figure 7 and Figure 8.

### High and low efficient ProSs

**Figure 6:**
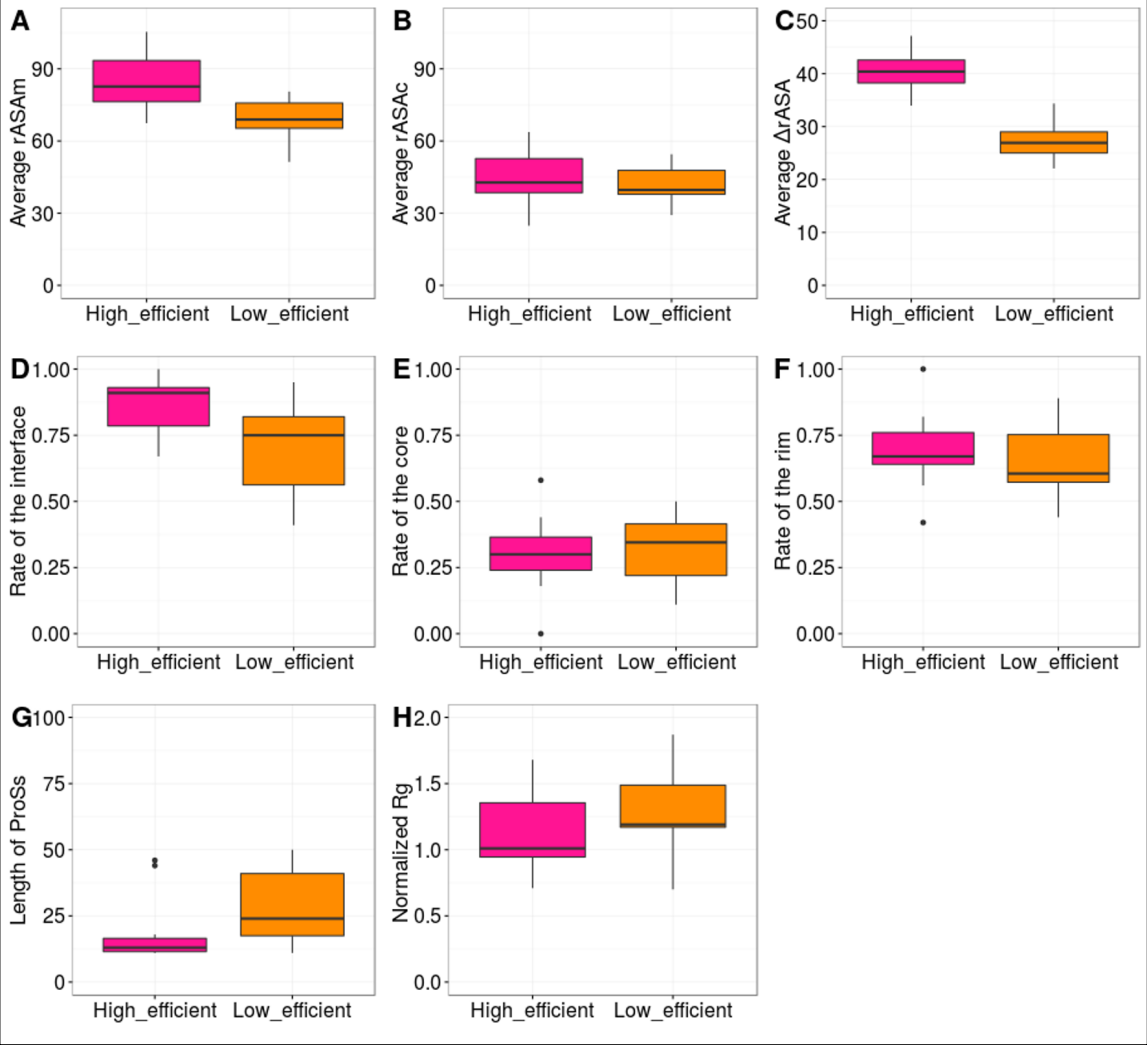
Box plots of the high and low efficient ProSs. The ProSs are classified into high and low efficient ProSs based on the average number of contacts at the interface. The high efficient ProS is shown in pink, and the low efficient ProS is shown in orange. The P-values are shown in Table 3.

**Figure 7:**
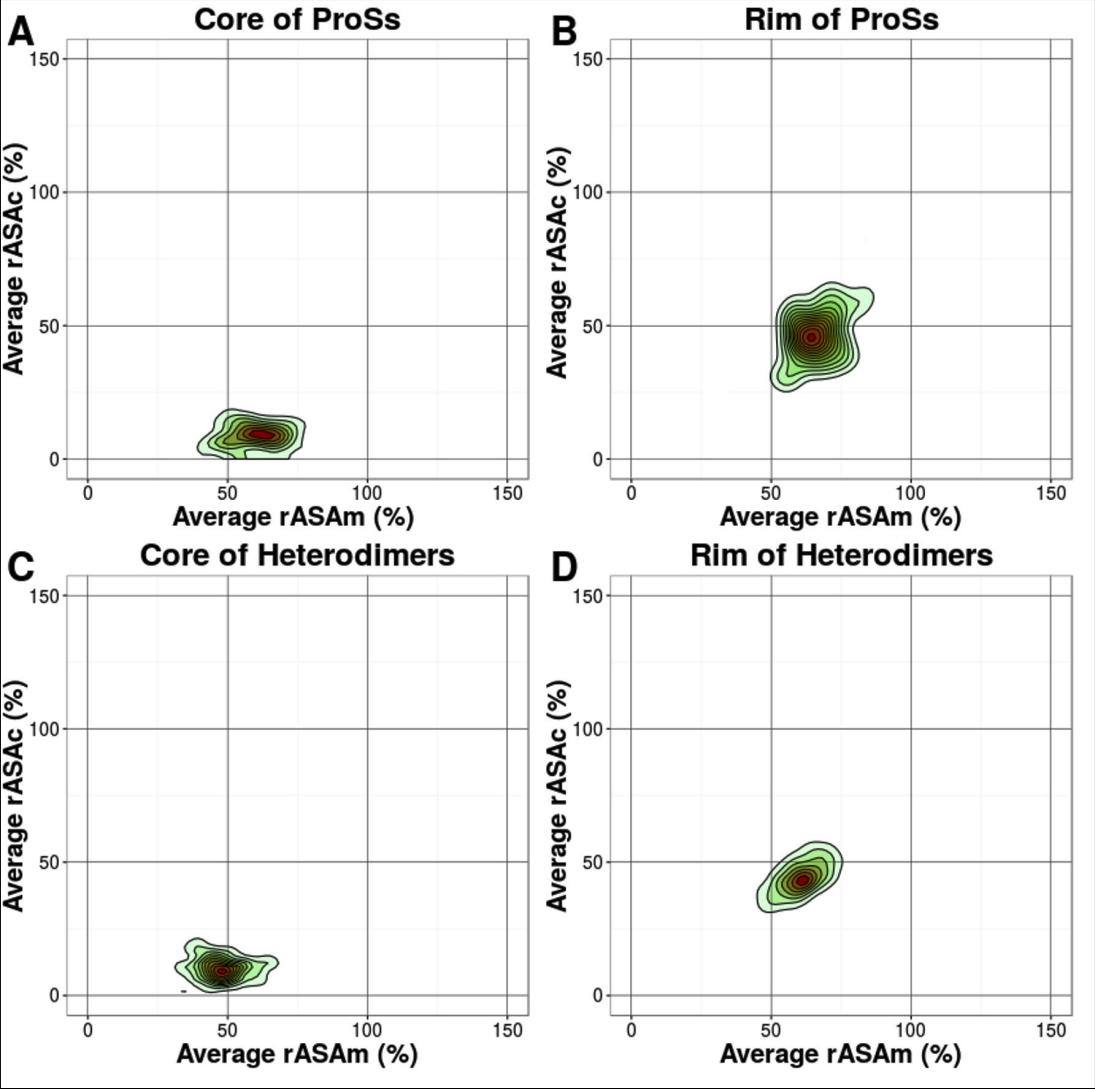
Contour plots of the average rASAm and average rASAc in alpha helices. (A) Average rASAm vs. average rASAc of the ProS core. (B) Average rASAm vs. average rASAc of the ProS rim. (C) Average rASAm vs. average rASAc of the heterodimer core. (D) Average rASAm vs. average rASAc of the heterodimer rim. The rASAs of each residue in the monomeric and in the complexed states in ProSs and heterodimers were calculated using Naccess [23]. The highest density regions are shown in red, and the lowest density regions are in green.

**Figure 8:**
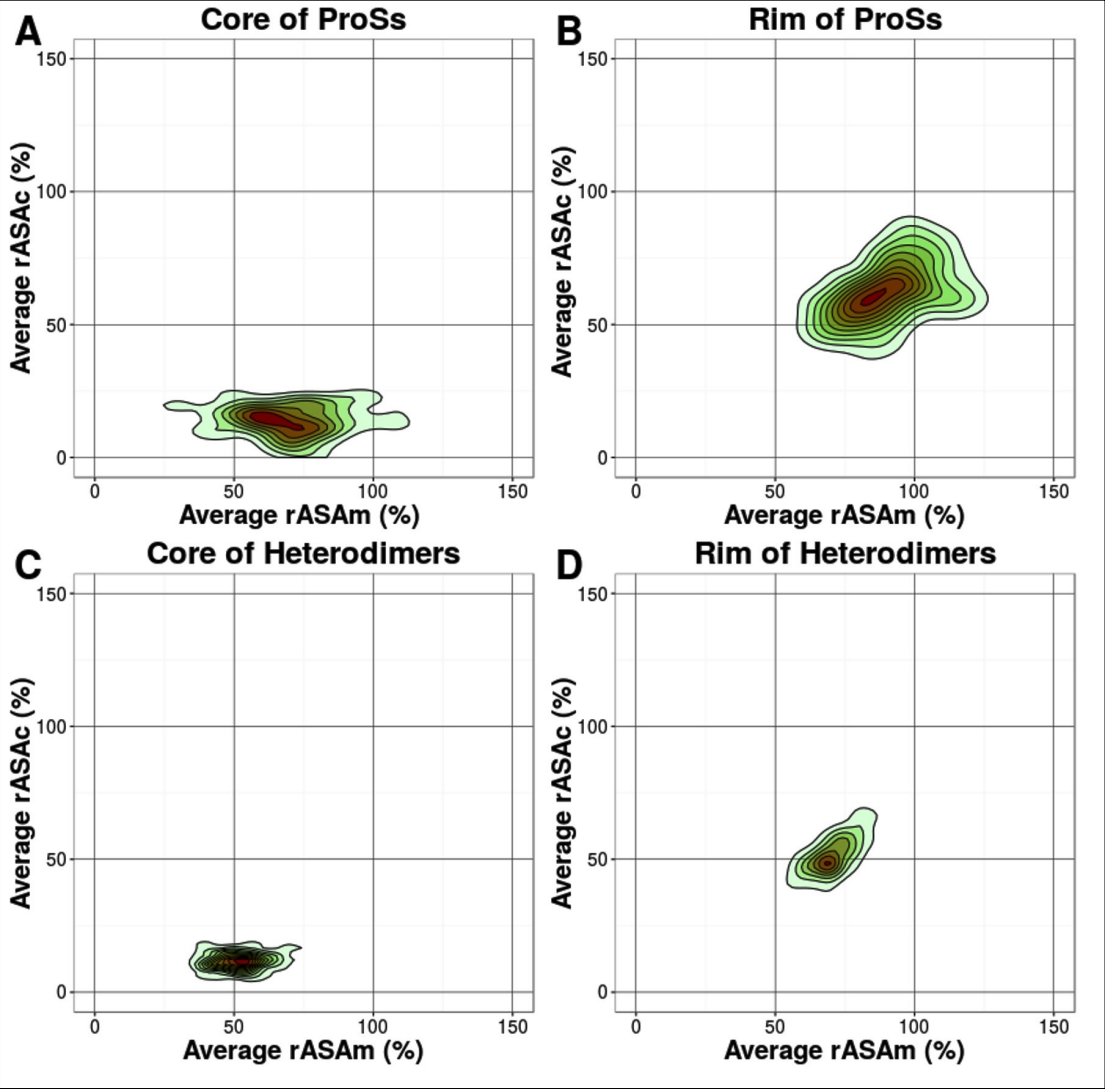
Contour plots of the average rASAm and average rASAc in irregulars. (A) Average rASAm vs. average rASAc of the ProS core. (B) Average rASAm vs. average rASAc of the ProS rim. (C) Average rASAm vs. average rASAc of the heterodimer core. (D) Average rASAm vs. average rASAc of the heterodimer rim. The rASAs of each residue in the monomeric and in the complexed states in ProSs and heterodimers were calculated using Naccess [23]. The highest density regions are shown in red, and the lowest density regions are in green.

Based on the average number of contacts at the interface, the ProSs were classified into high and low efficient ProSs (See Methods). To examine the properties of high efficient ProSs, several factors, such as average rASAm, average rASAc, average ΔrASA, rate of the interface, rate of the core, rate of the rim, radius of gyration (Rg), and length of the ProSs for each high and low efficient ProS were analyzed. Boxplots of the distributions of high and low efficient ProSs are shown in Figure 6 A-H . P-values of the high and low efficient ProSs are shown in Table 3.

The radius of gyration is used to describe the compactness of a protein, as well as the folding process from the denatured state to the native state [30,31]. The results show that there is no significant difference between the normalized radiuses of gyration (Rg) of high and low efficient ProSs. Similarly, the factors, such as average rASAc, rate of the interface, rate of the core, rate of the rim, and length of the ProSs are not statistically significant in both high and low efficient ProSs. The reason for this may be the low number of protean segments (ProSs) in the high and low efficient datasets. Interestingly, only the average rASAm and average ΔrASA are statistically significant. This confirms the hypothesis that average rASA in the monomeric state (rASAm) plays a major role in the efficient interactions of ProSs [13].

## Conclusion

The properties of secondary structure elements (SSEs) at the interface, core, and rim of ProSs were analyzed relative to those of heterodimers. The results demonstrate that irregular structures of ProSs and heterodimers are significantly different. Irregular structures have a larger rASA in the monomeric state (rASAm) that leads to the formation of many contacts in ProSs.

## Figures and Tables

**Table 1:** P-values of alpha helices in ProSs and heterodimers (using the Wilcoxon rank- sum test)

Features	Places	P-values
Average number of contacts	Interface	1.19E-18
	Core	3.47E-16
	Rim	0.0002
Average rASAm	Interface	3.26E-19
	Core	1.22E-13
	Rim	4.04E-05
Average rASAc	Interface	2.95E-05
	Core	0.008
	Rim	0.044
Average ΔrASA	Interface	1.15E-18
	Core	2.47E-14
	Rim	0.015

**Table 2:** P-values of irregular structures in ProSs and heterodimers (using the Wilcoxon rank- sum test)

Features	Places	P-values
Average number of contacts	Interface	2.36E-13
	Core	1.89E-09
	Rim	1.19E-16
Average rASAm	Interface	2.15E-44
	Core	4.15E-17
	Rim	1.80E-29
Average rASAc	Interface	3.52E-33
	Core	0.0009
	Rim	4.16E-18
Average ΔrASA	Interface	1.24E-14
	Core	9.79E-13
	Rim	5.21E-12

**Table 3:** P-values of high and low efficient ProSs (using the Wilcoxon rank-sum test)

Features	P-values
Average rASAm	0.0007
Average rASAc	0.403
Average ΔrASA	8.97E-07
Rate of the interface	0.028
Rate of the core	0.546
Rate of the rim	0.366
Length of the ProSs	0.02
Normalized Rg	0.228
